# Association of vectorcardiographic T-wave area with clinical and echocardiographic outcomes in cardiac resynchronization therapy

**DOI:** 10.1093/europace/euad370

**Published:** 2023-12-26

**Authors:** Muhammet Dural, Mohammed A Ghossein, Willem Gerrits, Fenna Daniels, Mathias Meine, Alexander H Maass, Michiel Rienstra, Frits W Prinzen, Kevin Vernooy, Antonius M W van Stipdonk

**Affiliations:** Department of Cardiology, Eskişehir Osmangazi University Faculty of Medicine, Odunpazarı, Eskişehir 26040, Turkey; Department of Cardiology, Cardiovascular Research Institute Maastricht (CARIM), Maastricht University Medical Centre+, Maastricht 6202, The Netherlands; Department of Physiology, Cardiovascular Research Institute Maastricht (CARIM), Maastricht University, Maastricht, The Netherlands; Department of Cardiology, University Medical Centre Utrecht, Utrecht, The Netherlands; Department of Cardiology, University of Groningen, University Medical Center Groningen, Groningen, The Netherlands; Department of Cardiology, University Medical Centre Utrecht, Utrecht, The Netherlands; Department of Cardiology, University of Groningen, University Medical Center Groningen, Groningen, The Netherlands; Department of Cardiology, University of Groningen, University Medical Center Groningen, Groningen, The Netherlands; Department of Physiology, Cardiovascular Research Institute Maastricht (CARIM), Maastricht University, Maastricht, The Netherlands; Department of Cardiology, Cardiovascular Research Institute Maastricht (CARIM), Maastricht University Medical Centre+, Maastricht 6202, The Netherlands; Department of Cardiology, Cardiovascular Research Institute Maastricht (CARIM), Maastricht University Medical Centre+, Maastricht 6202, The Netherlands

**Keywords:** Cardiac resynchronization therapy, T-wave area, QRS area, Heart failure, Bundle-branch block

## Abstract

**Aims:**

Data on repolarization parameters in cardiac resynchronization therapy (CRT) are scarce. We investigated the association of baseline T-wave area, with both clinical and echocardiographic outcomes of CRT in a large, multi-centre cohort of CRT recipients. Also, we evaluated the association between the baseline T-wave area and QRS area.

**Methods and results:**

In this retrospective study, 1355 consecutive CRT recipients were evaluated. Pre-implantation T-wave and QRS area were calculated from vectorcardiograms. Echocardiographic response was defined as a reduction of ≥15% in left ventricular end-systolic volume between 3 and 12 months after implantation. The clinical outcome was a combination of all-cause mortality, heart transplantation, and left ventricular assist device implantation. Left ventricular end-systolic volume reduction was largest in patients with QRS area ≥ 109 μVs and T-wave area ≥ 66 μVs compared with QRS area ≥ 109 μVs and T-wave area < 66 μVs (*P* = 0.004), QRS area < 109 μVs and T-wave area ≥ 66 μVs (*P* < 0.001) and QRS area < 109 μVs and T-wave area < 66 μVs (*P* < 0.001). Event-free survival rate was higher in the subgroup of patients with QRS area ≥ 109 μVs and T-wave area ≥ 66 μVs (*n* = 616, *P* < 0.001) and QRS area ≥ 109 μVs and T-wave area < 66 μVs (*n* = 100, *P* < 0.001) than the other subgroups. In the multivariate analysis, T-wave area remained associated with echocardiographic response (*P* = 0.008), but not with the clinical outcome (*P* = 0.143), when QRS area was included in the model.

**Conclusion:**

Baseline T-wave area has a significant association with both clinical and echocardiographic outcomes after CRT. The association of T-wave area with echocardiographic response is independent from QRS area; the association with clinical outcome, however, is not.

## Graphical Abstract

**Graphical Abstract:**
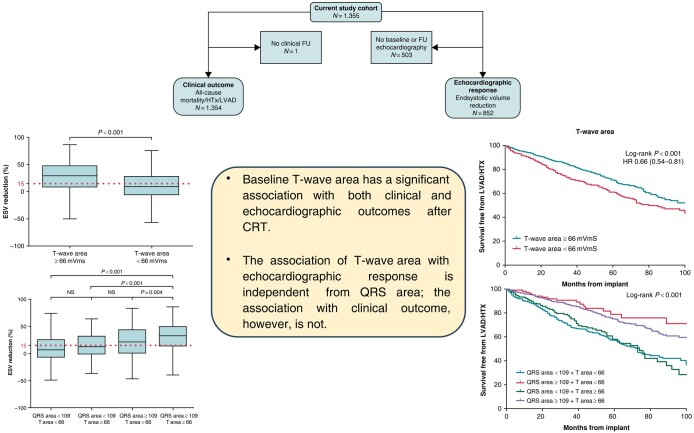

**What’s new?**

- Different left bundle branch block definitions and differences in clinical judgement encourage to study additional, less subjective electrocardiographic parameters to guide patient selection in cardiac resynchronization therapy (CRT).
- Data regarding the relation between vectorcardiographic T-wave area and CRT response on clinical outcomes are scarce. In addition, the relation between QRS area and T-wave area in CRT patients has not yet been assessed.
- Baseline T-wave area has a significant association with both clinical and echocardiographic outcomes after CRT.
- The association of T-wave area with echocardiographic response is independent from QRS area; the association with clinical outcome, however, is not.

## Introduction

Cardiac resynchronization therapy (CRT) is a proven treatment modality in heart failure (HF) with reduced ejection fraction (HFrEF) accompanied by ventricular conduction abnormalities. It has been shown that the response to CRT is better in those patients with a wide QRS and left bundle branch block (LBBB).^1–3^ Therefore, guidelines recommend that patient selection for CRT should be based on QRS morphology and duration.^4^ However, due to different LBBB definitions and differences in clinical judgement, significant variation in the classification of LBBB exists.^5^ Moreover, the use of these parameters entails a significant proportion of patients who experience little to no benefit from therapy despite being exposed to the risk of procedural and device-related complications. This encourages to study additional, less subjective electrocardiographic (ECG) parameters to guide patient selection in CRT.

Vectorcardiographic (VCG) QRS area has received a lot of attention as a new potential predictor of response to CRT.^6,7^ This VCG parameter, converted from a conventional 12-lead ECG, quantifies the extent and duration of unidirectional electrical depolarization and is potentially a better biomarker of the extent of electrical dyssynchrony of the left ventricle (LV) compared with QRS morphology and duration. However, repolarization is also significantly affected by the presence of ventricular dyssynchrony. The repolarization phase is influenced by the activity of many ion channels, especially those that determine the intracellular calcium concentration, which plays a role in contraction and relaxation.^8,9^ Moreover, in the process of HF, significant changes occur in the expression of many of these ion channels, including K^+^ and Ca^2+^.^10^ Disturbances in these ion channels may affect repolarization in dyssynchronous HF. Therefore, the changes in T-wave variables reflecting the plateau and repolarization phases of the action potential may provide additional information on ventricular dyssynchrony and CRT response. In a previous study, baseline T-wave area was found to be associated with LV ejection fraction (LVEF) increase following CRT in HF patients with LBBB.^9^ Subsequently, a large baseline T-wave area was shown to be a strong predictor of good clinical outcomes in CRT patients with LBBB.^11^ The results from these smaller, single-centre studies suggest that the T-wave area may provide additional value to the selection of patients most likely to benefit from CRT. However, data regarding the relation between T-wave area and CRT response on clinical outcomes are scarce. Moreover, the relation between QRS area and T-wave area in HF patients with LBBB has not yet been assessed.

In this study, we aimed to evaluate the association of baseline T-wave area with both clinical and echocardiographic outcomes of CRT in a large, multi-centre cohort of CRT recipients, and we evaluated the association between the baseline T-wave area and QRS area.

## Methods

For the current study, we analysed the Maastricht–Utrecht–Groningen (MUG) study cohort.^6^ This cohort retrospectively included all consecutive patients implanted with a CRT device in any of the three University Medical Centers in the Netherlands between January 2001 and January 2015. No formal inclusion or exclusion criteria were set on device, patient selection, or follow-up.

### Patient population

**Figure 1:**
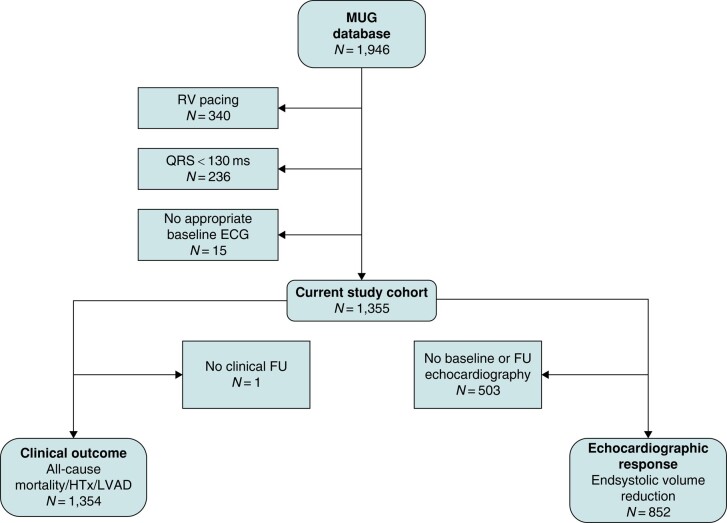
Patient data selection and availability for analyses. The entire MUG cohort consisted of all patients with a CRT device implanted from January 2001 to January 2015 in three university hospitals in the Netherlands. For the present study, patients with QRS < 130 ms and patients receiving an upgrade to biventricular pacing were excluded. Availability of data for analyses on the primary and secondary outcomes is also shown. ECG, electrocardiography; FU, follow-up; HTx, heart transplantation; LVAD, left ventricular assist device; RV pacing, right ventricular pacing.

The MUG cohort consists of 1946 patients with an available baseline 12-lead ECG. Patient selection for CRT implantation was done according to the prevailing guidelines.^12,13^ Patients with right ventricular pacing (340 patients; 17%) or QRS duration < 130 ms (236 patients; 12%) on their baseline ECG were excluded. An additional 15 (0.8%) patients in whom VCG analysis could not be performed due to frequent premature ventricular complexes were excluded. The patient selection is shown in *Figure 1*.

Baseline data were obtained from local hospital patient information systems. Clinical characteristics of patients such as HF cause and classification, medication, and comorbidity were retrieved from patient history and referral letters. If there was clear evidence of myocardial infarction, extensive coronary artery disease, or coronary artery bypass grafting as the underlying cause of the cardiomyopathy, the aetiology of HF was classified as ischaemic. Device parameters were obtained from specific device databases. Chest X-ray or fluoroscopic images were used to evaluate the LV lead location. The Dutch Central Committee on Human-Related Research [CCMO (Centrale Commissie Mensgebonden Onderzoek)] allowed the use of anonymous data without prior approval of an institutional review board provided that the data are acquired for routine patient care. All data used were handled anonymously. The study was designed according to the principles of the Declaration of Helsinki.

### Electro- and vectorcardiography

**Figure 2:**
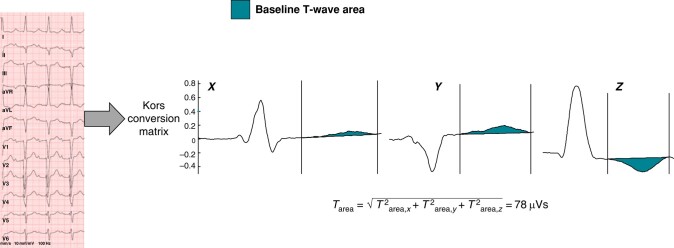
Transformation of ECG to VCG and calculation of baseline T-wave area. Twelve-lead ECGs are mathematically converted into VCGs with the three orthogonal *X*, *Y*, and *Z* leads using the Kors matrix. The *X*, *Y*, and *Z* leads of a patient before CRT are shown. T-wave area is then calculated from these three orthogonal leads using the formula presented.

Recorded baseline ECGs were digitally stored (MUSE Cardiology, GE Medical System) for T-wave area calculation as well as QRS area, QRS duration, and QRS morphology analysis. The ECGs up to 1 month prior to CRT implantation were included in the analysis. Automated ECG readings were used to evaluate the ECG parameters. QRS morphology was defined according to accepted criteria in the European Society of Cardiology (ESC) guidelines.^12^ For VCG analysis, the original digital signals were extracted from the digital PDF files stored in the MUSE system and converted from ECG to VCG automatically. For the T-wave area and QRS area calculation, custom MATLAB software (MathWorks Inc., Natick, Massachusetts) was used to convert the 12-lead ECG into three orthogonal VCG leads (*X*-, *Y*-, and *Z*-) using the Kors conversion matrix.^9,14^ The beginning and end of the T-wave and QRS complex were determined manually using the superimposed *X*, *Y*, and *Z* leads of the VCG. Thus, the area of the loops was analysed from the VCG. The T-wave area was calculated as the ‘three-dimensional’ areas between the curve and the baseline in the *X*, *Y*, and *Z* direction by using the following formula: (*T*^2^_area,*x*_ + *T*^2^_area,*y*_ + *T*^2^_area,*z*_)^1/2^ (*Figure 2*).^9,11^ QRS area was calculated similarly as the sum of the area under the QRS complex in the calculated VCG *X*, *Y*, and *Z* lead [QRS area = (QRS_area,*x*_^2^ + QRS_area,*y*_^2^ + QRS_area,*z*_^2^)^1/2^].

### Study outcomes

Echocardiographic outcome was the reduction in LV end-systolic volume (LVESV) determined by echocardiography between 3 and 12 months after implantation. Left ventricular ejection fraction and dimensions were preferably calculated by Simpsons modified biplane method. Echocardiographic response was defined as LVESV reduction ≥ 15%. If follow-up was not performed in the implantation centre, data were considered missing.

Clinical outcome was defined as the combination of all-cause mortality, heart transplantation (HTx), and LV assist device (LVAD) implantation. Data were obtained from hospital records linked to municipal registries for mortality data.

### Statistical analysis

The statistical analysis was performed using IBM SPSS statistics software version 28 (SPSS Inc.). Continuous and discrete variables are presented as mean ± SD and counts (percentages), respectively. Dichotomous variables were compared using Pearson’s *χ*^2^ test. Continuous variables were compared using a Student’s *t*-test. Stratification of T-wave area subgroups for presentation purposes and initial analyses was based on optimal binning with echocardiographic response as the determinant. Furthermore, the stratification of QRS area subgroups was based on optimal cut-off values found in binning analyses in previous studies^6,7^ using the same study population. Lastly, the study population was stratified according to the combination of T-wave area and QRS area, into four subgroups based on similar cut-offs. Kaplan–Meier survival analyses and cumulative hazard analyses were used when appropriate to evaluate the association between T-wave area and the clinical outcome. The log-rank test was used to determine the difference in survival probabilities between groups. Comparison of continuous echocardiographic (secondary) outcomes was performed using a one-way analysis of variance. Follow-up paired comparisons were made using the Tukey test. Cox and logistic regression analyses were used to assess univariable- and multivariable-adjusted effects of T-wave area on the association with the clinical and echocardiographic outcomes. Hazard ratio (HR) and odds ratio (OR) were reported, respectively. Multivariable regression analyses included covariates known to be associated with outcomes (clinical and echocardiographic) to CRT (including demographic, clinical, echocardiographic, device-type, and ECG parameters). QRS area was added to the model in a separate analysis to evaluate the additive value of T-wave area, next to QRS area. A two-sided *P*-value of <0.05 was considered statistically significant.

## Results

### Patient characteristics

The population represented a typical CRT population with ischaemic HF aetiology that was present in 49% of the patients and most of them were in New York Heart Association (NYHA) functional class II or III (93%). Mean QRS duration was 162 ± 19 ms and LBBB morphology was present in 80% of patients. The baseline characteristics of the patients are shown in *Table 1*.

**Table 1:** Baseline characteristics and *P*-values for statistical difference between baseline T-wave area groups

	All patients (*n* = 1370)	T-wave area (μVs) ≥ 66 (*n* = 794)	T-wave area (μVs) < 66 (*n* = 561)	*P*-value
Mean age (years)	67 ± 11	66 ± 11	67 ± 10	0.34
Women (%)	30	30	29	0.51
BMI, m/kg^2^	27 ± 5	26 ± 5	28 ± 5	0.30
Ischaemic CMP (%)	49	43	57	<0.001
Atrial fibrillation (%)	14	11	18	<0.001
Diabetes Mellitus (%)	25	22	29	0.006
Hypertension (%)	42	43	39	0.16
LVEF (%)	25 ± 9	24 ± 9	26 ± 9	0.77
LVEDV (mL)	220 ± 89	225 ± 92	211 ± 84	0.07
LVESV (mL)	168 ± 78	174 ± 81	159 ± 74	0.03
NYHA I (%)	2	3	2	0.03
NYHA II (%)	39	41	38	
NYHA III (%)	54	51	56	
NYHA IV (%)	5	5	4	
NT proBNP (pmol/L)	342 ± 610	363 ± 630	315 ± 583	0.26
CreatClear, mL/min	71 ± 32	72 ± 33	69 ± 31	0.13
Hb, mmol/L	8.5 ± 3	8.6 ± 3.5	8.4 ± 1	0.34
Beta-blocker (%)	82	82	82	0.94
ACEi/ARB (%)	90	90	91	0.57
MRA (%)	45	41	51	0.002
Upgrade (%)	14	12	17	0.007
CRT-D (%)	93	93	93	0.94
Appropriate lead position^a^ (%)	91	91	90	0.33
QRS duration (ms)	162 ± 19	168 ± 19	155 ± 16	0.005
LBBB morphology^b^ (%)	80	88	69	<0.001
T-wave area (μVs)	81 ± 41	106 ± 36	46 ± 13	<0.001

*P*-value was calculated using *χ*^2^ test. *P*-value below the alpha of 0.05 represents a statistical significant result.

ACEi, angiotensin-converting enzyme inhibitor; ARB, angiotensin receptor blocker; BMI, body mass index; CMP, cardiomyopathy; CreatClear, creatinine clearance; CRT-D, cardiac resynchronization therapy with defibrillation function; Hb, haemoglobine; LBBB, left bundle branch block; LVEDV, left ventricular end-diastolic volume; LVEF, left ventricular ejection fraction; LVESV, left ventricular end-systolic volume; MRA, mineral corticoid receptor antagonist; NT proBNP, N-terminal prohormone of brain natriuretic peptide; NYHA, New York Heart Association.

^a^Lateral or posterolateral lead positioning

^b^According to ESC guidelines.

Based on optimal binning with echocardiographic response as the determinant, the population was divided according to the optimal cut-off value of the baseline T-wave area before CRT implantation, resulting in a group of patients with a T-wave area ≥ 66 μVs (high T-wave area subgroup, *n* = 794) and a group of patients with a T-wave area < 66 μVs (low T-wave area subgroup, *n* = 561).

Patients in the high T-wave area subgroup showed a significantly lower rate of ischaemic HF aetiology (43% vs. 57%, *P* < 0.001), history of atrial fibrillation (11% vs. 18%, *P* < 0.001), and diabetes mellitus (22% vs. 29%, *P* = 0.006). Baseline LVESV (174 ± 81 mL vs. 159 ± 74 mL, *P* = 0.03), QRS duration (168 ± 19 ms vs. 155 ± 16 ms, *P* = 0.005), and the presence of LBBB morphology (88% vs. 69%, *P* < 0.001) were significantly higher in patients with a high T-wave area (*Table 1*).

### Clinical outcome

**Figure 3:**
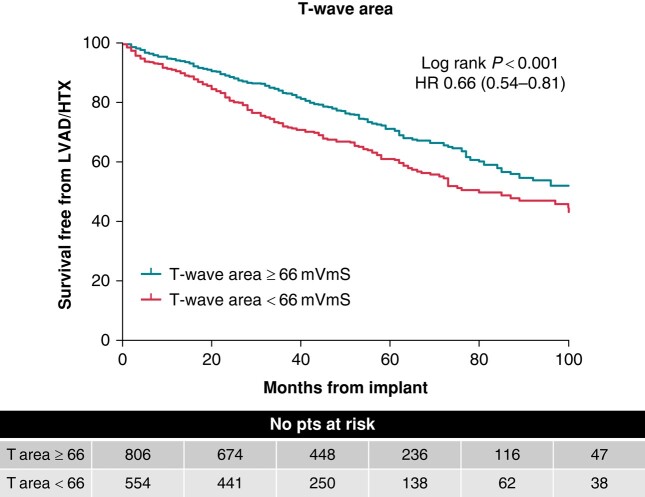
Kaplan–Meier estimates of survival free of the clinical outcome (combination of all-cause mortality, cardiac transplantation, and LVAD implantation). Patients are stratified by T-wave area of ≥ or <66 μVs. HR, hazard ratio; HTX, heart transplantation; LVAD, left ventricular assist device.

Data on the clinical outcome, the combination of all-cause mortality, HTx, and LVAD implantation, were available for 1354 patients (*Figure 1*). In total, 425 patients (31%) reached the clinical outcome with a mean follow-up time of 3.7 ± 2.9 years. The clinical outcome occurred significantly less in patients with a high T-wave area than in those with a low T-wave area [27% vs. 37%, HR 0.66 (0.54–0.81), *P* < 0.001). The Kaplan–Meier estimates of event-free survival in patient divided into subgroups based on baseline T-wave area are shown in *Figure 3*.

### Echocardiographic response

**Figure 4:**
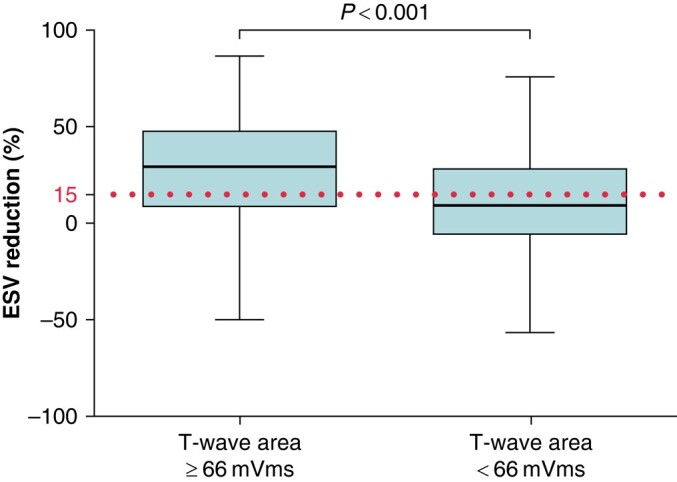
Echocardiographic reduction in LVESV and response rate. Echocardiographic LVESV reduction in percentage at follow-up echocardiography in patient groups stratified by T-wave area of ≥ or <66 μVs. Echocardiographic response was defined as ≥15% reduction of LVESV. LVESV, left ventricular end-systolic volume.

Baseline and follow-up LVESV measurements were available in 852 patients (63%). The mean time between implantation and echo was 6.6 ± 2.4 months. The mean reduction in LVESV for all patients was 19 ± 31%. Echocardiographic response to CRT, defined as LVESV reduction ≥ 15%, was seen in 487 (57%) of the 852 patients. Significantly more patients with high T-wave area were classified as echocardiographic responders than patients with low T-wave area (68% vs. 40%, respectively; OR: 3.1; confidence interval: 2.3–4.1, *P* < 0.001). In addition, mean LVESV reduction was significantly higher in patients with high T-wave area than in the low T-wave area subgroup (26 ± 30% vs. 9 ± 31%, *P* < 0.001, *Figure 4*).

### Regression analysis in relation to the clinical outcome and echocardiographic response

Multivariable regression analysis including ECG and clinical parameters showed that male sex, ischaemic HF aetiology, creatinine clearance, NYHA functional class, LVESV, and baseline T-wave area were associated with clinical outcome (*Table 2*). For the echocardiographic response, male sex, ischaemic HF aetiology, LBBB morphology, and T-wave area were the predictors (*Table 2*). Baseline T-wave area showed the strongest association with both the clinical outcome [HR 0.46 (0.31–0.69), *P* < 0.001] and echocardiographic response [OR: 3.1 (2.02–4.76), *P* < 0.001] (*Table 2*).

**Table 2:** Uni- and multivariable regression analyses for baseline T-wave area, ECG, and clinical parameters in relation to clinical outcome and echocardiographic response

	Univariable regression		Mulitvariable regression	
Clinical outcome (all-cause mortality, heart transplantation, LVAD)				
Variable	HR (95% CI)	*P*	HR (95% CI)	*P*
Male sex	1.81 (1.43–2.28)	<0.001	1.98 (1.27–3.10)	0.003
Age	1.02 (1.01–1.03)	<0.001	1.01 (0.98–1.03)	0.564
CRT-D	0.87 (0.59–1.29)	0.491		
Ischaemic CMP	1.53 (1.26–1.85)	<0.001	0.63 (0.42–0.92)	0.019
CreatClear	0.98 (0.98–0.99)	<0.001	0.98 (0.97–0.99)	<0.001
Beta-blocker	0.72 (0.57–0.90)	0.004	1.05 (0.66–1.68)	0.832
MRA	1.37 (1.06–1.77)	0.017	1.13 (0.79–1.61)	0.498
NYHA	1.89 (1.58–2.25)	<0.001	1.56 (1.15–2.13)	0.005
LVESV	1.00 (1.00–1.01)	<0.001	1.00 (1.00–1.01)	<0.001
Atrial fibrillation	1.72 (1.36–2.19)	<0.001	1.48 (1.00–2.28)	0.075
LBBB morphology^a^	0.57 (0.46–0.71)	<0.001	1.15 (0.75–1.77)	0.520
QRS duration ≥ 150 ms	0.77 (0.63–0.95)	0.015	0.72 (0.47–1.10)	0.119
T-wave area (μVs)	0.64 (0.53–0.78)	<0.001	0.46 (0.31–0.69)	<0.001

Echocardiographic response (ΔLVESV ≥15%)				
Variable	OR (95% CI)	*P*	OR (95% CI)	OR (95% CI)
Male sex	0.56 (0.41–0.75)	<0.001	0.61 (0.38–0.96)	0.033
Age	0.99 (0.98–1.00)	<0.092		
CRT-D	0.54 (0.30–0.97)	0.041	0.53 (0.25–1.12)	0.095
Ischaemic CMP	0.50 (0.38–0.66)	<0.001	0.59 (0.39–0.90)	0.013
Diabetes Mellitus	0.90 (0.65–1.24)	0.529		
Beta-blocker	1.03 (0.70–1.51)	0.886		
ACEi/ARB	1.14 (0.70–1.84)	0.607		
MRA	0.67 (0.47–0.95)	0.024	0.77 (0.51–1.15)	0.199
NYHA	0.79 (0.62–0.99)	0.044	0.77 (0.56–1.06)	0.110
LVESV	1.00 (0.99–1.00)	0.569		
Atrial fibrillation	0.51 (0.34–0.76)	<0.001	0.68 (0.38–1.21)	0.194
LBBB morphology^a^	2.96 (2.05–4.29)	<0.001	2.34 (1.37–4.00)	0.002
QRS duration ≥ 150 ms	1.91 (1.39–2.61)	<0.001	1.34 (0.83–2.18)	0.225
T-wave area (μVs)	3.15 (2.37–4.20)	<0.001	3.10 (2.02–4.76)	<0.001

*P*-value below the alpha of 0.05 represent a statistical significant result.

CI, confidence interval; CMP, cardiomyopathy; CreatClear, creatinine clearance; CRT-D, cardiac resynchronization therapy with defibrillation function; ECG, electrocardiography; HR, hazard ratio; LBBB, left bundle branch block; LVESV, left ventricular end-systolic volume; MRA, mineral corticoid receptor antagonist; NYHA, New York Heart Association; OR, odds ratio.

^a^According to ESC guidelines.

### T-wave area and QRS area

**Figure 5:**
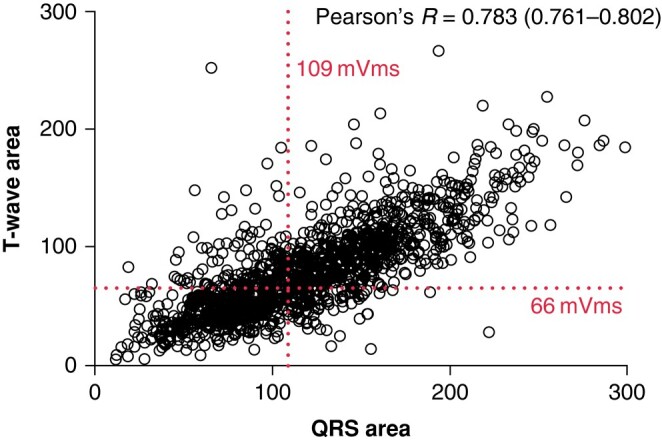
Scatter plot of the correlation between T-wave area and QRS area.

In accordance with previous studies, subgroups with baseline QRS area < 109 μVs^6^ showed a significant association with the clinical outcome. The risk of events was significantly lower in patients with high QRS area as compared with low baseline QRS area [HR 0.46 (0.38–0.56), *P* < 0.001]. Furthermore, echocardiographic response to CRT occurred significantly more often in patients with high QRS area than low QRS area [OR 3.8 (2.85–0.56), *P* < 0.001]. Interestingly, a significant correlation was found between T-wave area and QRS area (*P* < 0.001, *r* = 0.783) (*Figure 5*).

**Figure 6:**
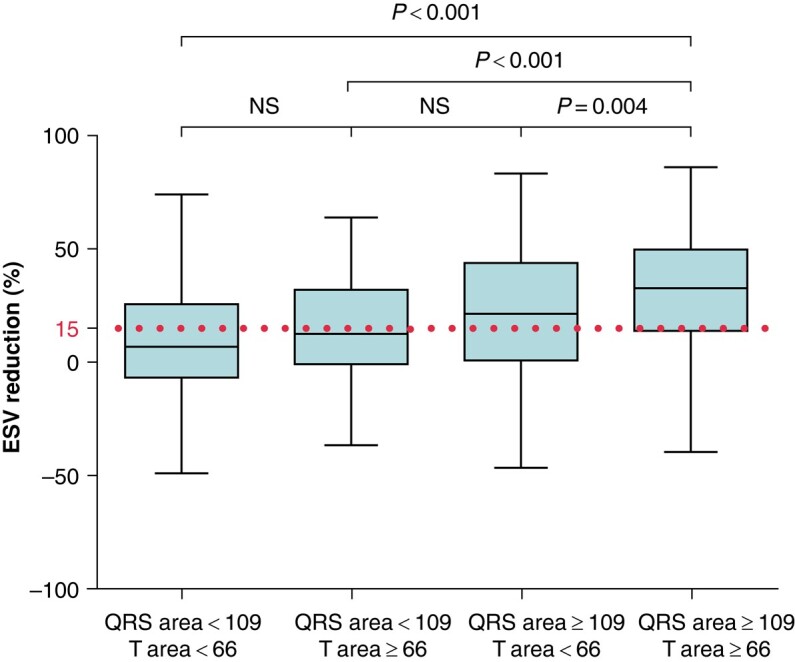
Echocardiographic reduction in LVESV and response rate according to QRS and T-wave area. Echocardiographic LVESV reduction in percentage at follow-up echocardiography in patient groups stratified by QRS area of < or ≥109 μVs and T-wave area of < or ≥66 μVs. Echocardiographic response was defined as ≥15% reduction of LVESV. LVESV, left ventricular end-systolic volume.

**Figure 7:**
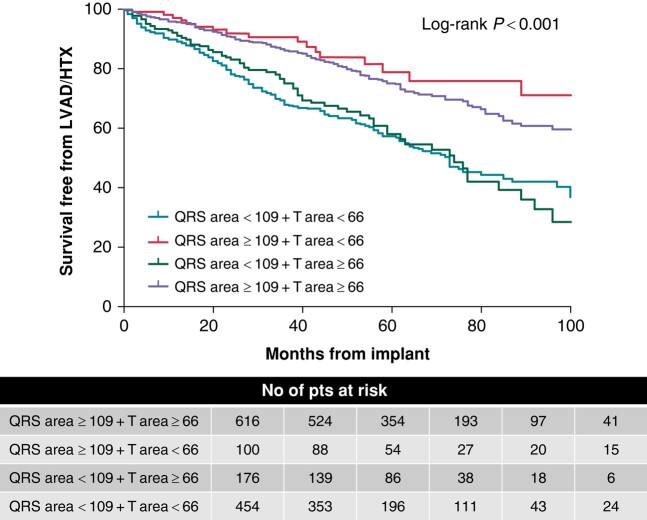
Kaplan–Meier estimates of survival free of the clinical outcome (combination of all-cause mortality, cardiac transplantation, and LVAD implantation) according to QRS and T-wave area. Patients are stratified by QRS area of < or ≥109 μVs and T-wave area of < or ≥66 μVs. HTX, heart transplantation; LVAD, left ventricular assist device.

When the patients were divided into four groups based on T-wave area (≥66 μVs vs. <66 μVs) and QRS area (≥109 μVs vs. <109 μVs), LVESV reduction was larger in patients with QRS area ≥ 109 μVs and T-wave area ≥ 66 μVs as compared with the other groups (*P* < 0.001) (*Figure 6*). Event-free survival rate was higher in patients with both QRS area ≥ 109 μVs and T-wave area ≥ 66 μVs [*n* = 616, HR 0.47 (0.38–0.58), *P* < 0.001], and patients with QRS area ≥ 109 μVs and T-wave area < 66 μVs [*n* = 100, HR 0.35 (0.21–0.56), *P* < 0.001], compared with the other two subgroups (*Figure 7*).

The addition of QRS area to the multivariable regression model showed that QRS area was associated with both the clinical outcome [HR 0.55 (0.33–0.93), *P* = 0.026] and the echocardiographic response [OR 2.2 (1.33–3.76), *P* = 0.002]. The association between the T-wave area and the echocardiographic response remained significant [OR 2.0 (1.20–3.36), *P* = 0.008] while the association with the clinical outcome did not persist [HR 0.68 (0.40–1.14), *P* = 0.143].

## Discussion

This large, multi-centre retrospective cohort study shows that baseline T-wave area is significantly associated with both the clinical and echocardiographic outcomes to CRT. This association is independent from known demographic, clinical, and ECG baseline determinants in CRT response. Even though T-wave area is significantly correlated to QRS area at baseline, it remains independently associated with echocardiographic response; it, however, does not with clinical outcome.

### Association of T-wave area with clinical and echocardiographic outcome to cardiac resynchronization therapy

Although there are many large studies examining depolarization-related ECG changes with CRT, data on repolarization parameters in CRT are scarce. Increased collagen content, loss of myofibrils, and gap junctional remodelling, which causes abnormal ventricular activation and electrical dyssynchrony, can also alter the repolarization pattern in patients with HF.^15–17^ Disturbances in ventricular repolarization can be evaluated by performing T-wave morphology analysis on 12-lead ECG.^18^ Some parameters derived from T-wave morphology analysis have been shown to be associated with adverse outcomes in patients with myocardial infarction and HF.^19,20^ While there are certain definitions for QRS morphology and duration, there are no clear criteria for T-wave morphology and duration. In many studies, T-wave properties were evaluated by combining them with other ECG parameters or determining the changes in duration, amplitude, and morphology from beat to beat or in a certain time interval.^21–23^ In a study, biventricular pacing has been shown to reduce T-wave alternans in tandem with reduced T-wave amplitude compared with right ventricular and LV pacing.^24^ Assessment of these parameters may be hard to implement in clinical practice. In each ECG lead, not only the T-waves follow the direction of the wide QRS complex, but also the whole T-wave three-dimensional vector aligns with the wide QRS vector when assessed using VCG.^25–27^ Therefore, it may be more appropriate to choose the VCG method to assess repolarization in this group of patients. T-wave area, which can be calculated quantitatively from the VCG, may provide important information about ventricular repolarization. Since T-wave area is a product of T-wave amplitude and T-wave duration, it can be assumed that a large T-wave amplitude and duration would also result in a larger T-wave area. However, little is known about its relation to clinical outcomes and reverse remodelling induced by CRT. In the study by Engels *et al*.,^9^ the relation between echocardiographic response at the sixth month and baseline ECG parameters was investigated in a medium-sized study of 244 CRT patients. Mean T-wave area was 84 ± 45 μVs and the increase in LVEF was larger in LBBB patients with T-wave area above the median value. Also, they found that a larger baseline T-wave area was associated with LVEF increase following CRT in patients with LBBB.^9^ Subsequently, the same investigators examined the relationship between baseline T-wave area and clinical outcomes in 335 CRT recipients.^11^ Patients reaching the primary composite endpoint of HF hospitalization, HTx, LVAD implantation, or death had a significantly smaller T-wave area (74 ± 45 μVs) compared with patients not reaching the primary endpoint (88 ± 47 μVs). They evaluated the patients by grouping them according to QRS morphology and T-wave area below or above the median value. During a mean 2.4-year follow-up period, the primary composite endpoint was significantly lower in the patient subgroup with a large T-wave area and LBBB than in patients with LBBB and a small T-wave area or non-LBBB patients with a small or large T-wave area.^11^ These findings are in line with the results from the present study. Considering the mean 3.7-year follow-up results of our study, containing 1354 patients, it was seen that primary outcomes occurred significantly less and echocardiographic response was larger in those with T-wave area ≥ 66 μVs. Additionally, the results of the present study show that T-wave area is an independent predictor of both clinical and echocardiographic outcomes. This study strengthened the results of previous studies by showing that baseline T-wave area is associated with both clinical and echocardiographic outcomes in CRT patients, with a large number of patients from different hospitals and a long follow-up duration.

### Combined evaluation of T-wave area and QRS area in response to cardiac resynchronization therapy

In recent studies, the QRS area appears to be a superior marker in the prediction of response to CRT, compared with QRS morphology and duration.^6,7,28^ As QRS area, representing the dispersion of the electrical depolarisation of the ventricles, and T-wave area, representing the dispersion of the repolarisation of the ventricles, represent different phases of the electrical cycle, these measurement might be complementary to each other in their association with outcomes in CRT patients. There are very few studies evaluating the QRS area and T-wave area together in CRT response. In our study, a total of 276 patients had baseline QRS area and T-wave area in the reverse direction, including 100 patients with high QRS area + low T-wave area and 176 patients with low QRS area + high T-wave area. When patients were stratified according to baseline QRS area and T-wave area, event-free survival was found to be significantly better in patient groups with baseline high QRS area + low T-wave area and high QRS area + high T-wave area, compared with the low QRS area + high T-wave area and low QRS area + low T-wave area, but no significant differentiation occurred within a QRS area of ≥109 and <109 subgroups by adding T-wave area cut-offs. Conversely, there was a significant difference between patient with baseline T-wave area of < and ≥66 within the QRS area of ≥109 group with respect to echocardiographic outcomes. T-wave area continued to be an independent predictor of echocardiographic response, but no longer was an independent predictor of clinical outcomes when QRS area was added to the model.

The study by Engels *et al*.^9^ showed that T-wave area is a predictor of echocardiographic response to CRT, and even better than QRS complex-related parameters, including QRS area, in LBBB patients. On the other hand, T-wave area had no predictive value in the non-LBBB subgroup and the relationship to clinical outcomes was not evaluated. Similar to our study, in the study of Vegh *et al*.^11^ investigating clinical response to CRT, they showed that T-wave area was an independent predictor of clinical response. However, QRS area was not included in this analysis. Also, the rate of patients with ischaemic HF, atrial fibrillation, and NYHA functional class III and IV was higher, while the rate of patients with LBBB was lower in their study as compared with our study. Unlike these studies, outcomes were examined by combining T-wave area with QRS area rather than QRS morphology in our study. Due to the different definitions of LBBB morphology, its use in clinical practice is not straightforward.^12,29,30^ Also, the LBBB classification by clinical judgement may show significant variability.^5^ From this perspective, VCG QRS area is a quantitative measurement and is more objective.

The results of our study show that patients with a high baseline T-wave area will benefit more from CRT if they also have a large QRS area. This may be explained by factors that are related to the variability in the relation between QRS and T-wave area. Further large-scale, prospective studies are needed to clarify the value of both T-wave and QRS areas in the prediction of response, before widespread adoption of these markers could improve patient selection in CRT.

### Limitations

Due to the retrospective design of our study, comparison with a non-treated group cannot be made. Therefore, it is not possible to allocate the difference in clinical outcomes to the effect of CRT. However, echocardiographic response is assessed using each patient as his/her own control and hence points to a clear association with the efficacy of resynchronization therapy and not a baseline difference in clinical prognosis, apart from amenability to CRT. In addition, due to the weak association between T-wave area and clinical outcome to get a binning value, stratification of T-wave area subgroups was based on its association with echocardiographic response. Moreover, response to CRT is affected by the patient's ECG and clinical characteristics, as well as the timing of the procedure and whether or not a defibrillator is present.^31,32^ Factors that have previously been associated with response to CRT, like time from first signs of HF to implantation, multipoint pacing, electromechanical coupling, and the overall improved outcomes in CRT over time, could not all be accounted for in the present analysis.^33–36^ It has been suggested that female hearts may show greater LV dyssynchrony than male hearts with the same QRS duration due to their smaller size.^37–39^ Other limitations of our study are that sex-related differences in outcomes were not evaluated and normalization of T-wave area for heart size was not performed. The retrospective nature limits the collection of these potentially confounding characteristics.

## Conclusions

This large population-based study demonstrates that baseline T-wave area has a significant association with both clinical and echocardiographic outcomes to CRT independent from baseline ECG determinants. Even though T-wave area remains associated with echocardiographic outcomes independent from QRS area, it does not with clinical outcomes. T-wave area, which is a simple and objective measurement, may contribute to patient selection for CRT, in addition to known parameters.

## Data Availability

Data are available from the corresponding author upon reasonable request.
